# A harmonized chemical monitoring database for support of exposure assessments

**DOI:** 10.1038/s41597-022-01365-8

**Published:** 2022-06-16

**Authors:** Kristin K. Isaacs, Jonathan T. Wall, Ashley R. Williams, Kevin A. Hobbie, Jon R. Sobus, Elin Ulrich, David Lyons, Kathie L. Dionisio, Antony J. Williams, Christopher Grulke, Caroline A. Foster, Josiah McCoy, Charles Bevington

**Affiliations:** 1 U.S. Environmental Protection Agency, Center for Computational Toxicology and Exposure, 109 T.W. Alexander Drive, Research Triangle Park, NC 27709 USA; 2 ICF International, 2635 Meridian Pkwy #200, Durham, NC 27713 USA; 3 U.S. Consumer Product Safety Commission 5 Research Place Rockville, Rockville, MD 20850 USA

**Keywords:** Environmental monitoring, Environmental impact

## Abstract

Direct monitoring of chemical concentrations in different environmental and biological media is critical to understanding the mechanisms by which human and ecological receptors are exposed to exogenous chemicals. Monitoring data provides evidence of chemical occurrence in different media and can be used to inform exposure assessments. Monitoring data provide required information for parameterization and evaluation of predictive models based on chemical uses, fate and transport, and release or emission processes. Finally, these data are useful in supporting regulatory chemical assessment and decision-making. There are a wide variety of public monitoring data available from existing government programs, historical efforts, public data repositories, and peer-reviewed literature databases. However, these data are difficult to access and analyze in a coordinated manner. Here, data from 20 individual public monitoring data sources were extracted, curated for chemical and medium, and harmonized into a sustainable machine-readable data format for support of exposure assessments.

## Abstract (summary)

**Table:** 

Measurement(s)	occurrence of chemicals in environmental and biological media
Technology Type(s)	mass spectrometry (curation of public data)
Sample Characteristic - Organism	Homo sapiens • aquatic invertebrates • aquatic vertebrates/mammals • birds • fish • terrestrial invertebrates/worms • terrestrial vertebrates • livestock
Sample Characteristic - Environment	surface water • drinking water • groundwater • ambient air • indoor air • indoor dust • landfill leachate • personal air • precipitation • wastewater • soil • sediment • sludge • consumer products • food products • raw agricultural commodities

## Background & Summary

Chemical exposure can be defined as the degree of contact between a chemical and a human or ecological target receptor (i.e., the person, population, or thing that is being exposed). EPA’s Exposure Forecasting (ExpoCast)^1^ project is charged with collecting exposure-relevant information for thousands of chemicals. This information feeds integrated datasets and predictive models that support risk-related decisions. The gold-standard method for quantifying occurrence to support exposure assessment is the analytical measurement of a chemical in the fluids or tissues of an organism (biomonitoring) or in an environmental medium such as air, water, or soil (environmental monitoring). These data, known collectively as chemical monitoring data, are used to assess exposures and ultimately risks in research and regulatory applications.

Despite their value, there are many challenges associated with the collection and use of chemical monitoring data. One key issue is that data are generated by many different government, academic, and commercial bodies, with each institution having unique methods of analysis and reporting. These differences contribute to variations in data quality and formatting, which complicates data synthesis. Chemical synonymy is a notable challenge; chemicals may be reported under many different names and associated identifiers including Chemical Abstract Service (CAS) Registry Numbers, European Community (EC) numbers etc., making it difficult to correctly assemble all related data for a given substance. Another notable challenge is data sparsity; biological and environmental monitoring studies are expensive and time consuming, and monitoring data simply do not exist for many chemicals used in commerce (tens-of-thousands). Many exposure assessments instead rely on predictive models that consider chemical uses, releases, and fate and transport. A recent report from the National Academies of Sciences on improving risk-related evaluations^2^ emphasizes the need to integrate measured and modelled data to improve confidence in exposure assessments.

The goal of the current effort is to develop a harmonized and well-curated (in terms of the specific chemicals and media in which they were monitored) database of chemical monitoring data to support predictive modelling efforts and efficient exposure assessments. This manuscript describes the collection and curation of a large amount of publicly available chemical monitoring data from various sources. The scope of the current effort includes data and reports made publicly available on the web by government agencies, academic groups, or others. A *de novo* search of the open literature was not performed here and is the focus of ongoing work. The general approach used in this study was to download (either manually or using standard scripting methods) individual data records and to compile them into a database containing both the raw data (i.e., stored using the original variable names from the individual sources) and a harmonized version. In the harmonized version the raw data were curated and assigned new standard variable names. The harmonized variables include, for example, information that describes the chemicals monitored; the media (i.e., the type of biological or environmental sample) in which chemicals were measured; temporal and geographic information; and analytical results including any reported concentrations, instrumentation information, detection/quantification limits, or quality assurance (QA) data.

These data have several potential uses. The first and primary use is to provide an accessible resource for drawing existing monitoring data into chemical assessments. The database described here provides a means to search existing data using standardized media names and chemical identifiers. This allows for the efficient development of geographic or temporal summaries when needed. In addition, the database will support development of data-driven exposure models. New data mining, cheminformatic, and machine-learning techniques have the potential to extract meaningful patterns from large chemical datasets. One initial application of the data described herein is the development of machine learning models for estimating the likelihood of occurrence of any chemical in a medium, based on the chemical’s structure and/or known use(s). The harmonized monitoring data provides a rich training set for such models. These data can also provide evaluation information for existing process-based models of chemical release, fate, and transport.

## Methods

The Multimedia Monitoring Database (MMDB) was compiled from existing reputable monitoring databases using a combination of automated and manual curation approaches. Datasets that are currently included in the database are listed in Table 1. These data sources were readily available monitoring databases that were reviewed to confirm that they met the following criteria:

1. *Accuracy-reliability* – Source is reputable, defined as government entity, or an entity with documented credentials regarding a particular topic.
2. *Applicability –* Source contains quantitative monitoring data for environmental samples and not spiked samples for the purpose of method validation or development.
3. *Representativeness* – Sample size must be greater than 5 measurements. Preference for data based on large surveys or studies as opposed to case studies or studies based on 1 or a few sites.
4. *Accessibility*- Datasets must be freely available, generally on publicly available websites, and preferably “FAIR” data^3^.

**Table 1:** Sources included in this Multimedia Chemical Monitoring Database.

Source	Abbreviation	Source Description	Website
American Healthy Homes Survey^12,13^	ahhs	Nationally-representative study of contaminants in homes by U.S. Department of Housing and Urban Development	https://www.epa.gov/ace/american-healthy-homes-survey-ahhs
National Atmospheric Deposition Program (Atmospheric Integrated Research Monitoring Network (AIRMoN)	airmon	AIRMoN is a monitoring network of seven sites in the Eastern U.S. - data were available for 1992–2015 (no	https://nadp.slh.wisc.edu/archived-networks/
Biomonitoring California	biomon_ca	Collaborative biomonitoring effort (The California Environmental Contaminant Biomonitoring Program, also known as Biomonitoring California), implemented by the California Department of Public Health and the California Environmental Protection Agency	http://biomonitoring.ca.gov/chemicals/chemicals-biomonitored-california
California Air Monitoring Network^14–19^	ca_airmon	Multi‐year air monitoring network to measure pesticides in various agricultural communities in California (2012–2016)	http://www.cdpr.ca.gov/docs/emon/airinit/air_network_results.htm
California Surface Water Database	ca_surf	Surface Water Database (SURF) maintained by the California Department of Pesticide Regulation (DPR), containing data from a wide variety of environmental monitoring studies	http://www.cdpr.ca.gov/docs/emon/surfwtr/surfdata.htm
California Air Resources Board (CARB)^20^	carb	Report from CARB to the California Legislature on indoor air pollution (2005)	https://www.arb.ca.gov/research/apr/reports/l3041.pdf
ChemTheatre	chem_theatre	ChemTHEATRE: Chemicals in the THEATRE [Tractable and Heuristic E-Archive for Traceability and Responsible-care Engagement], a platform for archival of environmental measurements supported by the Long-range Research Initiative (LRI) and the Japan Chemical Industry Association (JCIA)	http://chem-theatre.com/
Comparative Toxicogenomics Database^21,22^	ctd	A robust, publicly available database of data from published sources that aims to advance understanding about how environmental exposures affect human health	http://ctdbase.org/
EPA Nine POTW Study	epa_9potw	Results from an EPA Study of the occurrence of contaminants of emerging concern in wastewater from publicly owned treatment works (POTW)	https://www.epa.gov/sites/production/files/2018-11/documents/occurrence-cec-wastewater-9-treatment-work.pdf
U.S. Environmental Protection Agency (EPA) Ambient Monitoring Technology Information Center – Air Toxics Data	epa_amtic	Ambient Monitoring Archive of the EPA’s Ambient Monitoring Technology Information Center (AMTIC) The archive covers measurements of hazardous air pollutants (HAPS) from as early as 1990 to 2016. The archive for HAPs currently houses data from over 2,500 monitoring sites.	https://www3.epa.gov/ttnamti1/toxdat.html
EPA Discharge Monitoring Report Data	epa_dmr	State-level data for 2007–2016 from discharge monitoring reports from EPA’s Enforcement and Compliance History Online site.	https://cfpub.epa.gov/dmr/
EPA Office of Water, National Study of Chemical Residues in Lake Fish Tissue^23^	epa_nscrlft	Data from a published report on a national EPA study to estimate the national distribution of selected persistent, bioaccumulative, and toxic (PBT) chemical residues in fish tissue from lakes and reservoirs of the United States.	https://www.epa.gov/sites/default/files/2018-11/documents/national-study-chemical-residues-lake-fish-tissue.pdf
Targeted National Sewage Sludge Survey^24^	epa_tnsss	2009 EPA survey to examine over 350 pollutants in sewage sludge.	https://www.epa.gov/biosolids/sewage-sludge-surveys
EPA Unregulated Contaminant Monitoring Rule	epa_ucmr	Data collected under the EPA Unregulated Contaminant Monitoring Rule (UCMR3). The rule is used to collect data for contaminants that are suspected to be present in drinking water and do not have health-based standards set under the Safe Drinking Water Act (SDWA). State-level data from 2013–2015.	https://www.epa.gov/dwucmr/occurrence-data-unregulated-contaminant-monitoring-rule
U.S. Food and Drug Administration (FDA) Total Diet Study	fda_tds	Ongoing FDA program that monitors levels of about 800 contaminants and nutrients in the average U.S. diet. Database includes data from 2003–2011.	http://www.fda.gov/Food/FoodScienceResearch/TotalDietStudy/ucm184293.htm
ICES-DOME	ices	Marine Environment Data Portal of The International Council for the Exploration of the Sea (ICES), an intergovernmental marine science organization.	https://www.ices.dk/data/data-portals/Pages/DOME.aspx
Information Platform for Chemical Monitoring Data (IPCHEM)	ip_chem	IPCHEM is a web single access point for locating and accessing chemical monitoring data across all media in the European Union. Data included both environmental and biomonitoring data.	https://ipchem.jrc.ec.europa.eu/RDSIdiscovery/ipchem/index.html
National Health and Nutrition Examination Survey	nhanes	National Health and Nutrition Survey. 2018 Fourth National Report on Human Exposure to Environmental Chemicals. Updated Tables, March 2018, Volume One.	https://stacks.cdc.gov/view/cdc/53006
U.S. Department of Agriculture (USDA) National Residue Program (NRP)	usda_nrp	Chemical residue results for meat, poultry, and egg products.	https://www.fsis.usda.gov/science-data/data-sets-visualizations/residue-chemistry
United States Geological Service (USGS) Monitoring Data –National Water Quality Monitoring Council	usgs	Monitoring data from USGS for air, biological tissue, groundwater, sediment, soil, surface water, and tissue (2010–2018).	http://www.waterqualitydata.us/portal/

The sources may be further refined (e.g., by media type or other data subset) in later tables. Details of these sources (including extraction method and additional links) are provided in Supplementary Table S1.

Data sources were categorized as either “single-sample”, where each record was a single analytical measurement, or “summary” (or “aggregate”), where each record was a summary metric (e.g., a mean, median, or specific percentile) for a group of measurements.

Data were collected from these data sources in three data collection phases (dictated by EPA funding cycles and generally occurring during the years 2017, 2018, and 2019). Some sources were simply updated in the 2018 and 2019 cycles and while some sources were newly added. Both automated and manual processes were used to curate unique data sources into the format of the multimedia monitoring database. Each data source was unique in format. Where necessary, data were obtained from the original sources using R or Python scripts. Biomonitoring data for the IPCHEM data source were provided directly by the IPCHEM team in CSV format. For data sources in PDF form, a combination of manual extraction and automated extraction was used to generate the dataset (method varied depending on source; all methods and scripts were retained). In sources that had tables that could be directly exported, the data were saved and manually reformatted to CSV format. Detailed descriptions of the source data, including the number of records and location/availability of metadata, the original form of raw data, type of data extraction (e.g., manual or script) and phase(s) of data collection are provided in Supplementary Table S1.

**Fig. 1:**
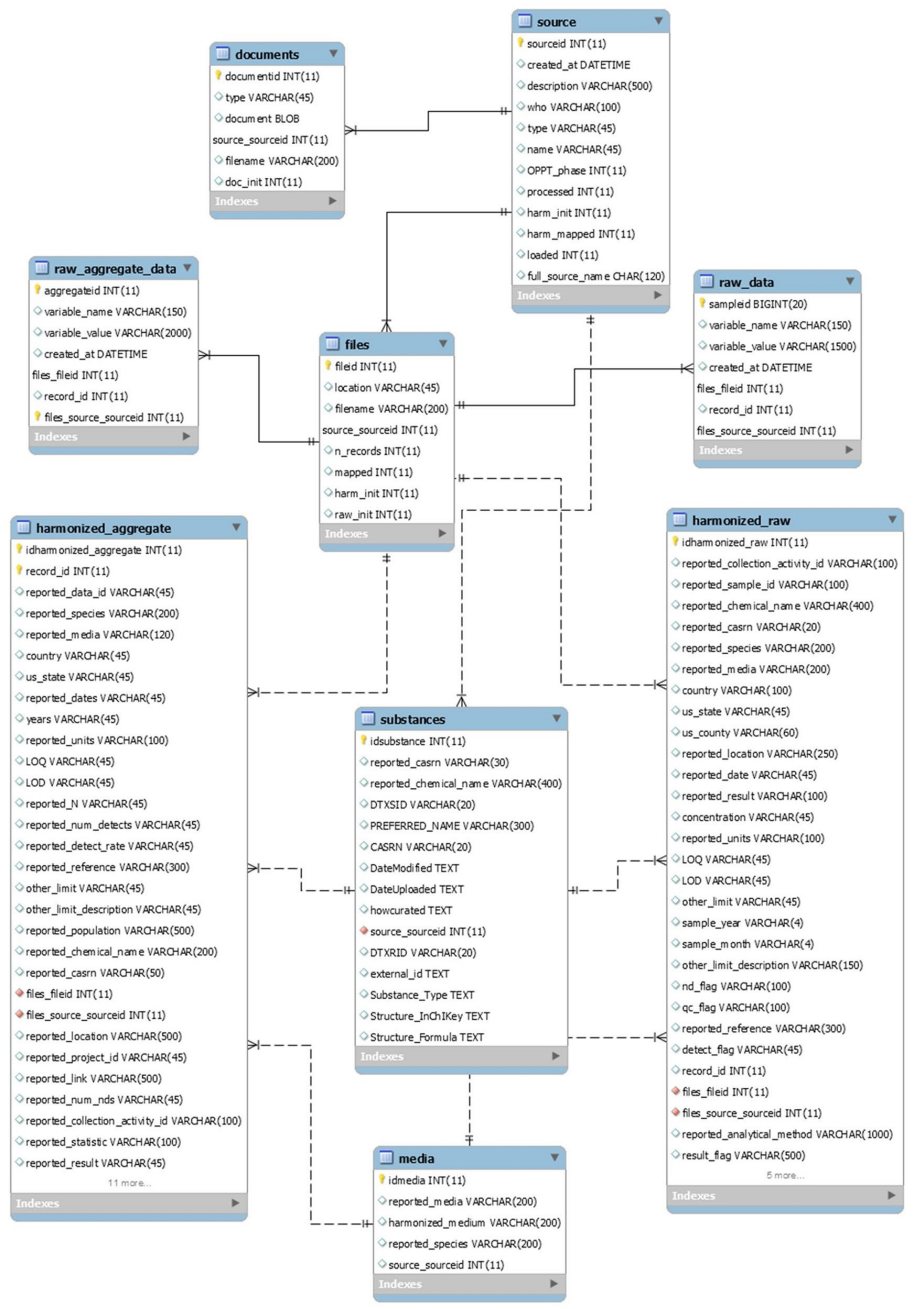
MMDB Entity Relationship Diagram. See Supplementary Table S2 for a full description of database variables.

**Fig. 2:**
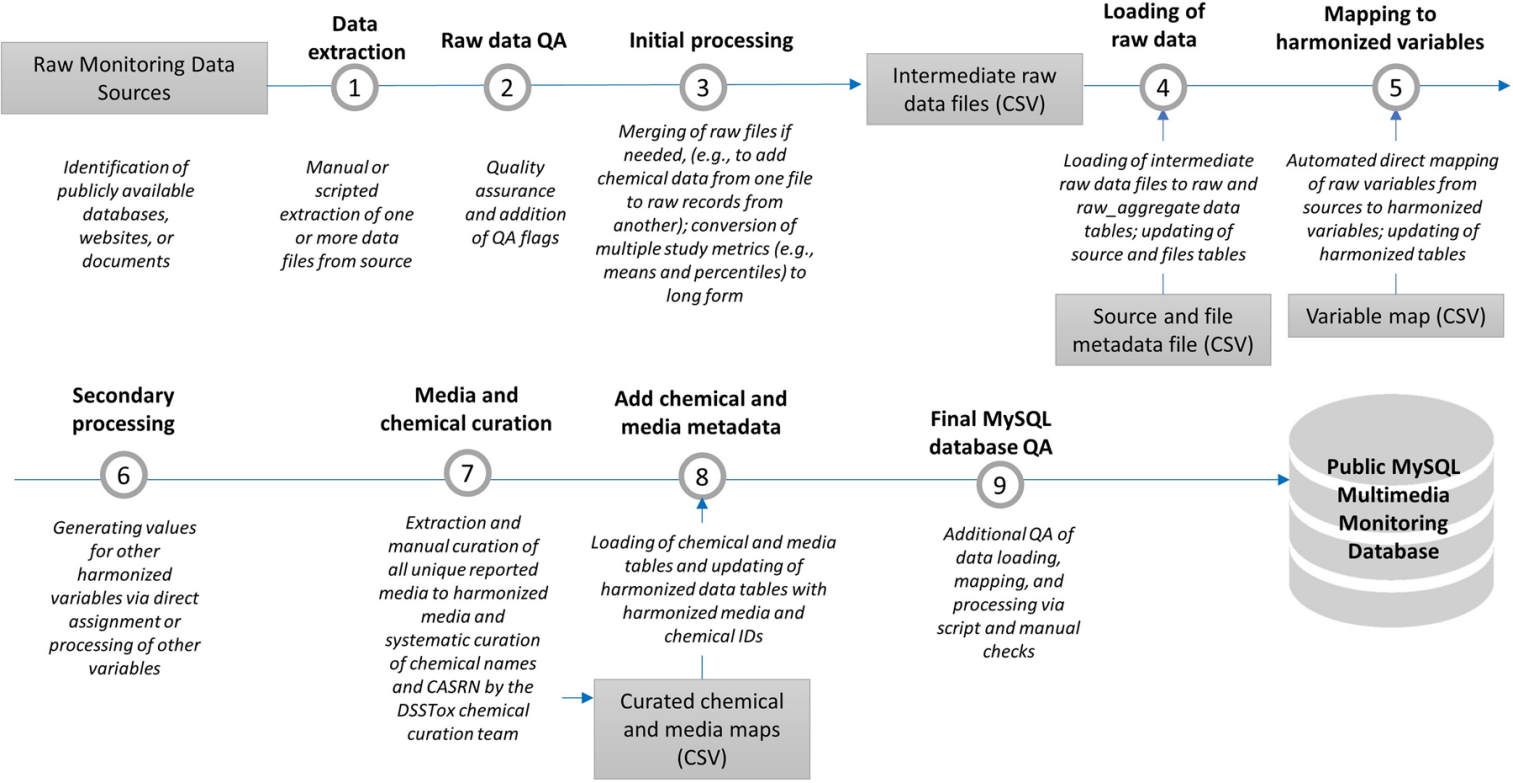
Workflow for creating the Multimedia Monitoring Database. Details of each workflow phase are included in the Methods and Technical Validation sections.

A MySQL^4^ relational database was designed to store both the raw and harmonized data and source metadata. The database entity-relationship diagram is provided in Fig. 1. All tables are described below in Data Records; all variable and table definitions are provided in Supplementary Table S2. The general workflow for populating the database is shown in Fig. 2 and described in detail below. In brief, raw data extracted from the data sources were pre-processed, loaded into the database, and then harmonized to standard variable names via an automated mapping process. Media and chemical identifiers were then also harmonized and secondary variables generated. Quality assurance (QA) of the raw data and the final database is described in the Technical Validation section.

### Data extraction

Data were extracted manually, via script, or through direct download, dependent on the data source. Details per database and per primary reference, where appropriate, are provided in Supplementary Table S1. Online databases often required multiple queries to obtain data efficiently and files were concatenated or pre-processed to join various outputs. Details of queries and pre-processing are provided for each source in the “Data and Curation Details” field in Table S1.

### Initial processing

The raw data files underwent initial processing to prepare them for loading into the raw data tables of the database. In some cases, metadata from the raw source that would facilitate curation (e.g., chemical identifier information) were included in separate raw data files or downloads. In these cases, files were merged on appropriate raw variables (e.g., an internal chemical ID) included in both files. In the case of summary studies, it was desirable to organize the data into single variable “reported statistic” (e.g., a “mean” or “median”) and a “value” of that rather than a wide table of many metrics. This standardized the format across the raw summary studies and simplified later processing and reporting. Therefore, the raw data were “melted” using tools in the “reshape” package within the R environment prior to loading. Note that this step does not remove or alter any of the raw data, rather it just reshapes it into an optimal form. Note that aggregated summary statistics reported in MMDB were as reported in the original data sources (none were calculated by the authors); different sources may have handled values under the limit of quantification (LOQ) differently and thus the original source metadata should be consulted. Flags were added to the database (where possible) to indicate records associated with values <LOQ.

### Loading of raw data

Once the intermediate raw data files (CSV) were extracted, quality checked, and processed, they were loaded into MMDB using an R script and an input control file. The control file contains information about every data source including directory and file paths for the intermediate raw data files. First, a unique entry for the data source was created in the source table, with accompanying source ID, name, data type (e.g., single-sample or summary), and other identifiable data. Next, the unique file information (e.g., filename, directory location, row count) for each data source’s raw data files were added to the files table, with relational reference to the source ID from the source table. Once file information was prepped, the file data were transformed into long form (to standardize storage) and written to the corresponding raw data table based on data source type. The raw data, from both single-sample data summary sources, were fed into a raw data table in this long form, with each record containing a variable name and a value, to allow storage of all original source data regardless of number of variables or format. Each row entry was also tagged with its corresponding file ID, source ID, and record ID. Finally, if a data source has additional supplemental or data documents, their file information was loaded into the documents table in a similar manner as for the file table. At every loading step, binary indicator variables track if a specific step was completed. This ensures that if a script fails or has an error, the workflow can pick up exactly where it left off. This also saves computational resources if a new data source is added, deleted, or updated.

### Mapping to harmonized variables

Once the raw data source files were loaded to the raw table, their variables and values were ready to be transformed into a harmonized form across data sources. This was performed using an R script and data source variable map. The variable map, given in Supplementary Table S3, is a file containing every raw data field from each data source, mapped to the corresponding harmonized variable names. First, empty fields were added to the corresponding harmonized table for a data source, by data type, based on the file ID, source ID, and record ID within the raw table. Next, the raw data were transformed back into wide form. Then, both the raw variable name values and the mapped name values were processed to unify character case, white space, and remove punctuation, to ensure mapping was not inhibited, before the raw variable names were renamed for harmonization. The harmonized raw data, now in wide form, was then written to the harmonized table with accompanying source ID, file ID, record ID, and new harmonized ID.

### Secondary processing

After the raw data were harmonized, further secondary processing was performed to enrich data analysis. That is, additional harmonized variable values were created by further processing raw variables (e.g., populating a flag indicating the value was a non-detect from a character appended to a raw analytical result) or entering study metadata (e.g., adding a reported medium of “ambient air” for an ambient air-related data source). Key additions were location variables and a detection indicator variable. Location data were unified into “country”, “US State”, and “US County” fields from the harmonized tables’ “reported location” field. For most, this was a duplicate field, while others required mapping from country abbreviations, country codes, or sampling site ID. A particularly useful harmonized variable was a flag indicating whether an observation was associated with detection of the chemical (if it could be discerned from available data). For the detection variable, an R script was created to assign a 1 or 0 value to a harmonized record based on the “reported result”, “LOD”, “LOQ”, and other QA flags. Additional variables derived from secondary processing may be added in the future.

### Media and chemical extraction and curation

The original reported chemical substances and media underwent curation efforts to harmonize them for modeling. To assist with harmonizing chemicals and media, a unique list of values from each source was extracted from the harmonized tables. This included reported chemical name and CAS number, and reported media and reported species, for chemical and media data respectively. The reported chemical names and CAS Registry Numbers (CASRN) were curated into EPA’s Distributed Structure-Searchable Toxicity (DSSTox) Database using an automated curation workflow described elsewhere^5^. Prior to automated curation, parenthetical names were parsed to provide an additional name for potential automapping. The resulting curation process produced a chemical list including unique identifiers (DSSTox substance identifier, DTXSID) for each substance. The automated mapping process assigns a DTXSID with a given QC flag level (indicating confidence in the curation). Whether or not a given record was curated depended multiple factors including the previous confirmed curation of the identifier into DSSTox and assignment to substance IDs to the identifier. Not all identifiers will have corresponding IDs; a common case in this database were measurements associated with mixtures. Manual curation of the list of the identifiers included in this dataset by a trained curation team is ongoing as resources allow. The reported sample media (e.g., a species and tissue name or water sample type) were mapped to a set of 32 unique media (listed in Table 2). Mappings of all reported chemicals and media identifiers to their harmonized values are provided in Supplementary Tables S4 and S5. Once mapped, the unique values were written to the substance and media tables with unique substance and media ID values. In addition, observations in the harmonized tables not associated with chemical measurements were removed, as these were sometimes reported in the same fields as chemical measurements in various data sources. This included many environmental condition or weather measurements in the USGS data source, and physiological measurements on studied species in the ICES data source.

**Table 2:** Harmonized media identifiers in the multimedia monitoring database.

Harmonized Medium	Description
**Environmental**	
ambient air	Outdoor ambient air
drinking water	Treated or untreated drinking water supplies, tap water, bottled drinking water, cooking water
groundwater	Water from groundwater sources (wells, aquifers)
product	Non-food consumer products
sediment	Freshwater or marine sediments
sludge	Sewage sludge
soil	Soil, sand, or outdoor settled dust
surface water	Lake, river, or marine surface water; includes rainwater
indoor air	Residential or other indoor air samples
indoor dust	Residential or other indoor dust samples (from any location)
landfill leachate	Landfill leachate (water having passed through landfill solids)
other-environmental	Other environmental media, not classified elsewhere
personal air	Personal air sample or exhaled breath
precipitation	Snow, rainfall, or other atmospheric deposition
wastewater (influent, effluent)	Inflow or outflow samples from municipal or industrial sites
**Human Biomonitoring**	
breast milk	Human breast milk
human (other tissues or fluids)	Human tissues or fluids other than blood or urine, including nails, hair, semen, adipose tissue, saliva, sputum, sweat, amniotic, fluid, bone, and others
human blood (whole/serum/plasma)	Human whole blood, blood cells, serum, plasma, or other extractants, including fetal or umbilical samples
urine	Human urine
skin wipes	Wipes from human skin (any body surface)
**Wildlife Biomonitoring and Edible Foods**	
wildlife (aquatic invertebrate)	Marine or freshwater invertebrates (e.g., crustaceans, mollusks etc.), any tissue
wildlife (aquatic vertebrates/mammals)	Non-fish aquatic vertebrates or mammals, any tissue
wildlife (birds)	Avian species, any tissue (including eggs)
wildlife (fish)	Fish species, any tissue
wildlife (terrestrial invertebrates/worms)	Terrestrial invertebrates, any tissue
wildlife (terrestrial vertebrates)	Terrestrial vertebrates, any tissue
other-ecological	Other ecological species not categorized elsewhere, including algae and seaweeds
vegetation	Terrestrial vegetation including non-processed fruits and vegetables
livestock/meat	Unprocessed meat products or samples from non-fish animals to be used as food
raw agricultural commodity	Unprocessed raw fruits, vegetables, grains, nuts, or seeds that have been grown for food
food product	Processed food products, including dairy products, breads, cooked meats, processed (e.g., canned or frozen) fruit and vegetable products, infant formula

Records were assigned to “unknown” if the medium could not be determined from the reported information.

## Data Records

The monitoring data compiled and harmonized here are stored in a MySQL relational database maintained by the USEPA and available via Figshare^6^. An export of the MySQL database is archived; this file contains a set of SQL statements that can be executed to reproduce the original database object definitions and all table data. Users may install MySQL and download the file from Figshare for manipulation and data extraction. Addition of new data, updating of harmonized variables from raw variables, and updates to the underlying monitoring dataset are ongoing. Versioned updates of the database will be provided as available, e.g., as more raw chemical identifiers in the raw data are curated or as reported concentration data are curated to standard formats and units. Details presented below represent data records archived in the MySQL database V1.0.

Within the MySQL database, there are tables containing data records and tables including metadata. The data records may be raw monitoring data from the original source or harmonized data records. Metadata tables contain descriptions and information which relate to all data records (or large subsets), as compared to record-specific data which is specific to a single data record (e.g., a single analytical measurement or study metric). Metadata may include information about individual data sources or downloaded files, or information about the chemicals or media referenced in the data tables. Metadata tables are linked to the data record tables by a set of database IDs referencing a specific data source, downloaded raw file, medium, or chemical.

There are 9 tables in the MySQL database. A full description of all variables included in the database tables is included in Supplementary Table S2.

**Fig. 3:**
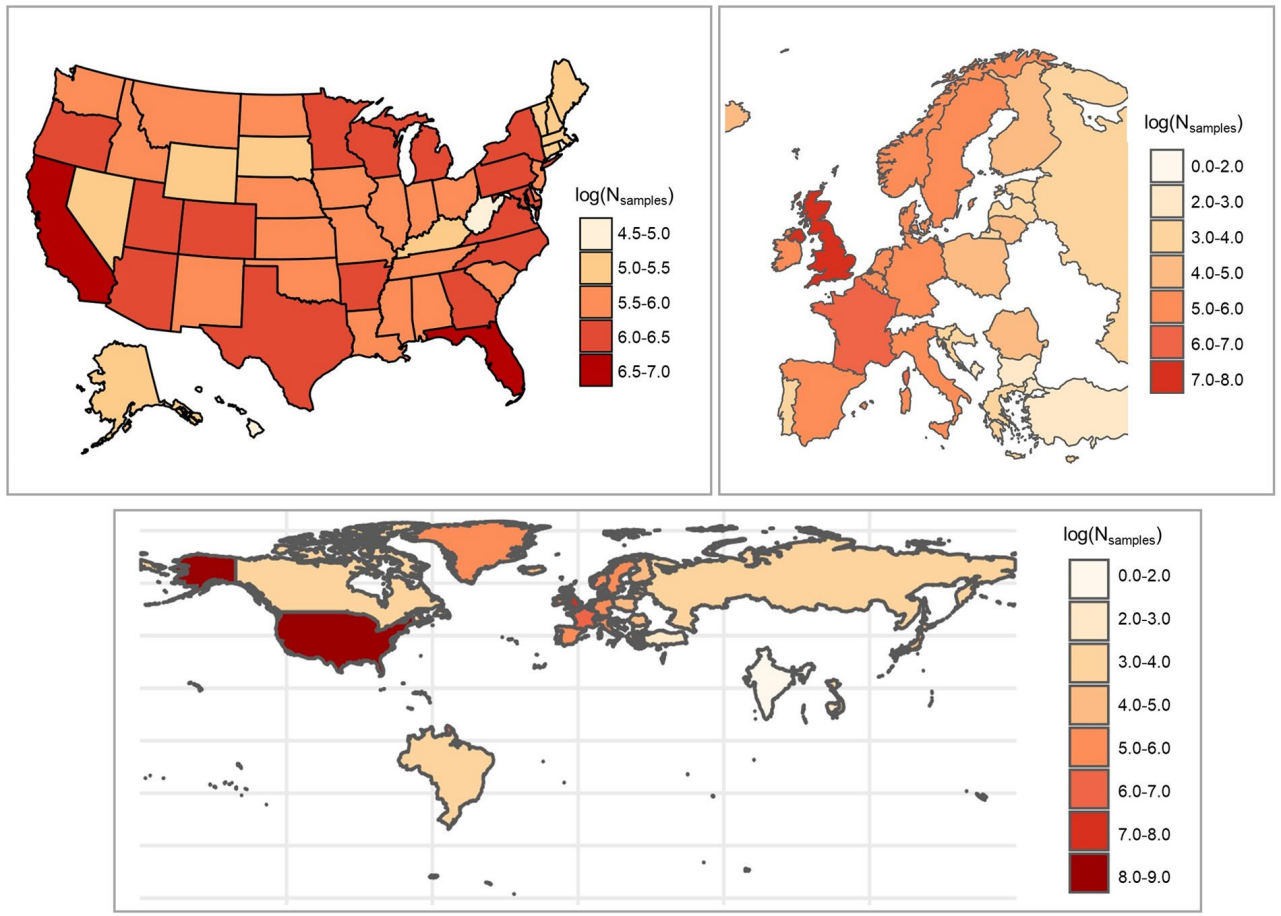
Location of origin of single-sample data in the Multimedia Monitoring Database. Single-sample data are from 42 countries, with most samples from the United States or Europe. Color denotes count of individual samples (N_samples_) in each country or U.S. state.

The multimedia database contains 63,768,583 individual harmonized data records (54,520,407 single-sample records and 9,248,176 aggregate records.) A total of 9,956 unique raw reported chemical (substance) identifiers (name and/or CASRN) were identified; 8,757 these could be mapped in the DSSTox database to one of 3,271 unique DTXSIDs. The mapped chemicals represent a wide range of chemical substance and use classes, including metals, pesticides, flame retardants, polychlorinated biphenyls, pharmaceuticals, and both consumer and industrial use chemicals. Counts of database observations (single-sample and summary) associated with each curated DTXSID are included in Supplementary Table S6. A summary of the chemicals and media represented in each of the database sources is given in Table 3. Figure 3 provides a summary of the location (US State, European Country, and worldwide occurrence) associated with each single-sample record in the database; counts of samples in countries outside the U.S. and Europe were small compared to these locations.

**Table 3:** Summary of chemicals, media, and data by source.

Data Type	Source	Unique Curated DTXSIDs	Unique Chemical Identifiers	Media Represented	Number of Observations
Summary	ahhs	29	29	indoor dust; soil	57
Single-sample	airmon	9	9	ambient air	342540
Summary	biomon_ca	91	92	human blood (whole/serum/plasma)	2616
Summary	ca_airmon	41	44	ambient air	452
Single-sample	ca_surf_sediment	120	123	sediment	72205
Single-sample	ca_surf_water	362	380	surface water	497463
Summary	carb	10	11	ambient air; indoor dust; personal air	368
Single-sample	chem_theatre	424	498	wildlife (fish); wildlife (aquatic vertebrates/mammals); wildlife (terrestrial vertebrates); wildlife (birds); sediment; wildlife (terrestrial invertebrates/worms); surface water; soil; wastewater (influent, effluent); vegetation; unknown; ambient air; groundwater	49058
Summary	ctd	801	906	unknown; product; human (other tissues or fluids); ambient air; personal air; indoor air; wildlife (fish); skin wipes; food product; raw agricultural commodity; human blood (whole/serum/plasma); wildlife (aquatic invertebrate); indoor dust; soil; livestock/meat; breast milk; vegetation; sediment; surface water; urine; wastewater (influent, effluent); drinking water; groundwater	100826
Single-sample	epa_9potw	172	176	wastewater (influent, effluent)	3150
Single-sample	epa_amtic	91	217	ambient air	2871688
Summary	epa_dmr	825	1332	wastewater (influent, effluent)	4111611
Summary	epa_nscrlft	196	231	wildlife (fish)	3696
Single-sample	epa_tnsss	143	145	sludge	12181
Single-sample	epa_ucmr	33	35	drinking water	1036486
Single-sample	fda_tds_elem	19	35	raw agricultural commodity; food product	142365
Single-sample	fda_tds_pest	150	252	raw agricultural commodity; food product	20100
Single-sample	ices_biota	330	447	wildlife (fish); wildlife (aquatic vertebrates/mammals); wildlife (birds); wildlife (aquatic invertebrate); vegetation; wildlife (terrestrial vertebrates)	1262673
Single-sample	ices_sediment	303	391	sediment	533236
Single-sample	ip_chem_biomonitoring	137	176	human (other tissues or fluids); urine; wildlife (fish); vegetation; wildlife (terrestrial vertebrates); wildlife (aquatic invertebrate); wildlife (terrestrial invertebrates/worms); human blood (whole/serum/plasma); sediment; wildlife (birds)	182761
Single-sample	ip_chem_biota	74	99	wildlife (aquatic invertebrate); wildlife (fish); other-ecological; vegetation; wildlife (birds); wildlife (aquatic vertebrates/mammals); wildlife (terrestrial vertebrates)	826827
Summary	ip_chem_ibs	14	17	urine	4216
Summary	ip_chem_lakes	689	832	surface water	4761124
Single-sample	ip_chem_seawater	85	155	surface water	350160
Single-sample	ip_chem_sediment	82	112	sediment	338066
Summary	nhanes	244	444	human blood (whole/serum/plasma); urine	84665
Single-sample	usda_nrp	45	49	livestock/meat; food product	5051
Single-sample	usgs	2154	2852	surface water; wildlife (fish); sediment; ambient air; groundwater; wastewater (influent, effluent); wildlife (aquatic invertebrate); wildlife (terrestrial invertebrates/worms); precipitation; wildlife (terrestrial vertebrates); soil; unknown; other-environmental; other-ecological; drinking water; vegetation; landfill leachate; wildlife (aquatic vertebrates/mammals); livestock/meat; wildlife (birds)	46152942

Source subsets defined in Supplementary Table S1. For summary sources, the observations include different summary statistics for each chemical.

## Technical Validation

The main quality objectives for the multimedia database were to ensure that data included in the raw data tables accurately reflected the data as provided in the raw data source, and that variables and data were accurately mapped to harmonized variables. Efforts to curate the dataset have not focused on checking for errors that may have been made by the original provider of the data; however, QA of raw data for suspected issues and QA of the harmonized tables was performed. Organizations or individuals who use this database are encouraged to perform QA consistent with data quality objectives for their assessments.

### Raw data quality assurance

Raw datasets were divided into two categories: (i) tabular datasets and (ii) manually extracted data from an existing report or pdf. For tabular data, an R script was developed that reviewed each line of data within the set to determine if it met criteria to be flagged for a specific QA concern. The script appended the QA concern to the row of data if it met one or any of the criteria for any of the QA flags. One row of data could have multiple QA concerns associated. Separate R scripts were developed for each dataset, since they contained different variable names, however the scripts were based on the same QA flag criteria. An independent reviewer ran the R script and verified (i) proper file selection and loading of files via file name, row counts, and column counts and (ii) appropriate assigned of column headers to variables used in flagging code.

Counts for each individual flag created by the code were conducted. Datasets that were small enough to be opened in Excel were filtered by various variables in Excel to determine a manual count of the number of rows expected to be flagged for each QA concern. For datasets that exceeded the data limit in Excel, logic in R was used to obtain a count of the expected number of rows of data to be flagged for each QA concern. These independent, expected flag counts were then compared to the number of rows flagged by the QA R script to determine if the R script accurately flagged the correct number of rows. If there was a discrepancy, the script was reviewed and revised until the counts matched.

For manually extracted datasets, data were extracted from the original source into a standard Excel template with standard variable names. The datasets were then independently reviewed to verify the transcription accuracy. An R script was developed to append the QA codes to the extracted data (see Supplementary Table S7). The resulting flagged datasets were then reviewed by an independent reviewer to confirm the script identified all QA concerns accurately.

For datasets where the sampling year, unit of measurement, or media was missing, a manual review of the database documentation was performed to determine if the missing data could be identified. If the missing information could be identified, it was added to the dataset through the R script.

### QA of harmonized data

Data entries within all database tables were checked in an automated QA process using three separate code scripts. These scripts checked key points in the workflow for data loading and processing. The first checked for missing and extraneous data source ID, file ID, and document ID values for associated table row entries. It also checked if the correct number of data records matched between the raw files and raw and harmonized tables. The script reported any discrepancies found between the raw CSV files and raw data table. The second checked for harmonized variable mappings between raw and harmonized tables. The third checked the mapping between media and chemical map tables to harmonized table entries. The output of this script could be visually inspected for any obvious errors in mapping (especially in the media mappings). The mappings of chemical records from raw chemical identifiers to DTXSID were obtained using an established semi-automated chemical curation workflow. Mappings are assigned QC levels based on the method and confidence of the mapping. Refinement of algorithms used in the semi-automated mapping process, and additional manual verification of mappings by the trained DSSTox curation team are always ongoing.

## Usage Notes

MMDB is currently released as a MySQL “dump” file containing MySQL Statements that can be used to recreate the database objects (e.g., tables) and all data. MySQL is a free, open-source database management system (DBMS). It can be installed on Windows or Linux machines and provides both a server application (MySQL server) for creating, updating, and hosting databases (such as MMDB) and client application (MySQL client) for querying functionality. Once MySQL is installed and configured, the MMDB MySQL file can be run using MySQL server to create an exact copy of the original database. Note that when uncompressed, the MMDB file is very large (over 300GB), as is the database, so this may be a limitation on some systems. Once the database is built, a user can query MMDB using standard SQL commands (e.g., using MySQL client) or other scripting methods. The R programming language has packages that provide a direct interface to MySQL (“RMySQL”) and allow one to query a MySQL database (and transform the data) using simple syntax (“dplyr”). An example R script for querying MMDB by chemical or media using the RMySQL and dplyr packages is provided by the authors (see Code Availability).

The MySQL DBMS system and our robust standardized data loading and mapping procedures support the straightforward addition of other sources of monitoring data in the future. We further note that the database will be supported and maintained (as resources allow) under EPA’s Chemical Safety for Sustainability Research Program. In the future, it is planned that this data or summaries from MMDB will be incorporated into EPA ORD’s data infrastructure and be surfaced via the CompTox Chemicals Dashboard^7^ (https://comptox.epa.gov/dashboard) or other public-facing systems as ORD continues to work to make exposure-relevant data accessible to stakeholders. In addition, curation of the existing data, for updating or addition of other useful variables (e.g., flags indicating handling of non-detects in original summary sources) or harmonization of reported concentrations to standardized units, may continue.

Though data included in the database can be used in many ways for future analysis, users should be aware of limitations of the dataset, and appropriate usage of the data. Although a wide range of chemicals are included in the database, all the data here are the result of targeted analytical studies, wherein one or more chemicals were identified *a priori* for inclusion. Recent suspect-screening and non-targeted analysis of environmental and biological samples provides evidence that the true number of man-made or naturally-occuring chemicals present in biological and environmental media may be greater than what is currently included in this database. Therefore, this database does not contain an exhaustive list of chemicals found in organisms or the environment. In addition, there are still a significant number of raw chemical identifiers present that have not been mapped to harmonized identifiers (for several reasons, including their absence from the DSSTox database of synonyms for known substances or the inability to be considered a single chemical substance (e.g., mixtures of co-eluting PCBs). However, the raw reported identifiers are included in the database (and listed in Supplementary Table S4) and thus could be further addressed by end-users.

Due the size of this database, it is expected to provide a useful training set for machine-learning models that predict the occurrence of chemicals in different media. Other chemical information, such as chemical structure, chemical properties, and information about how chemicals are used are potential descriptors for such models. These models will complement analogous models developed by the ExpoCast project which predict chemical functional use^8,9^ and exposure pathway^10^. Within the ExpoCast project, new efforts are underway to identify thousands of chemicals present in environmental media using new non-targeted analytical (NTA) methods^11^. This database and subsequent models built upon it provide a useful resource for confirming tentative identifications in NTA studies.

## Supplementary information

Table S1.

Table S2.

Table S3.

Table S4.

Table S5.

Table S6.

Table S7.

## Data Availability

All scripts used to obtain raw data, clean or process raw data, perform QA, and construct the database are available in the MMDB Processing Scripts folder at 10.23645/epacomptox.16674298. Various versions of R and python were used in different project stages; the primary version for both data cleaning and building the database was R version 3.6.2. An example R script containing sample queries of MMDB by chemical and media is maintained in the Sample Queries folder. We will also maintain an SQL script (to be run in MySQL immediately after the MMDB dump file) to correct any identified curation mistakes in official MMDB releases in the MMDB Correction Scripts folder.
